# Impact of peer support on virologic failure in HIV-infected patients on antiretroviral therapy - a cluster randomized controlled trial in Vietnam

**DOI:** 10.1186/s12879-016-2017-x

**Published:** 2016-12-16

**Authors:** Do Duy Cuong, Anders Sönnerborg, Vu Van Tam, Ziad El-Khatib, Michele Santacatterina, Gaetano Marrone, Nguyen Thi Kim Chuc, Vinod Diwan, Anna Thorson, Nicole K. Le, Pham Nhat An, Mattias Larsson

**Affiliations:** 1 Global Health, Department of Public Health Sciences, Karolinska Institutet, Stockholm, Sweden; 2 Infectious Diseases Department, Bach Mai Hospital, Hanoi, Vietnam; 3 Department of Medicine Huddinge, Karolinska Institutet, Stockholm, Sweden; 4 Division of Clinical Virology, Department of Laboratory Medicine Huddinge, Karolinska Institutet, Stockholm, Sweden; 5 Vietnam-Sweden Uong Bi General Hospital, Quang Ninh, Vietnam; 6 World Health Programme, Université du Québec en Abitibi-Témiscamingue (UQAT), Rouyn-Noranda, Canada; 7 Unit of Biostatistics, Institute of Environmental Medicine, Karolinska Institutet, Stockholm, Sweden; 8 Hanoi Medical University, Hanoi, Vietnam; 9 Morsani College of Medicine, University of South Florida, Tampa, FL USA; 10 Oxford University Clinical Research Unit (OUCRU), Hanoi, Vietnam

**Keywords:** Peer support, Virologic failure, Antiretroviral therapy, HIV, CD4 count, Vietnam

## Abstract

**Background:**

The effect of peer support on virologic and immunologic treatment outcomes among HIVinfected patients receiving antiretroviral therapy (ART) was assessed in a cluster randomized controlled trial in Vietnam.

**Methods:**

Seventy-one clusters (communes) were randomized in intervention or control, and a total of 640 patients initiating ART were enrolled. The intervention group received peer support with weekly home-visits. Both groups received first-line ART regimens according to the National Treatment Guidelines. Viral load (VL) (ExaVir™ Load) and CD4 counts were analyzed every 6 months. The primary endpoint was virologic failure (VL >1000 copies/ml). Patients were followed up for 24 months. Intention-to-treat analysis was used. Cluster longitudinal and survival analyses were used to study time to virologic failure and CD4 trends.

**Results:**

Of 640 patients, 71% were males, mean age 32 years, 83% started with stavudine/lamivudine/nevirapine regimen. After a mean of 20.8 months, 78% completed the study, and the median CD4 increase was 286 cells/μl. Cumulative virologic failure risk was 7.2%. There was no significant difference between intervention and control groups in risk for and time to virologic failure and in CD4 trends. Risk factors for virologic failure were ART-non-naïve status [aHR 6.9;(95% CI 3.2–14.6); *p* < 0.01]; baseline VL ≥100,000 copies/ml [aHR 2.3;(95% CI 1.2–4.3); *p* < 0.05] and incomplete adherence (self-reported missing more than one dose during 24 months) [aHR 3.1;(95% CI 1.1–8.9); *p* < 0.05]. Risk factors associated with slower increase of CD4 counts were: baseline VL ≥100,000 copies/ml [adj.sq.Coeff (95% CI): −0.9 (−1.5;−0.3); *p* < 0.01] and baseline CD4 count <100 cells/μl [adj.sq.Coeff (95% CI): −5.7 (−6.3;−5.4); *p* < 0.01]. Having an HIV-infected family member was also significantly associated with gain in CD4 counts [adj.sq.Coeff (95% CI): 1.3 (0.8;1.9); *p* < 0.01].

**Conclusion:**

There was a low virologic failure risk during the first 2 years of ART follow-up in a rural low-income setting in Vietnam. Peer support did not show any impact on virologic and immunologic outcomes after 2 years of follow up.

**Trial registration:**

NCT01433601.

## Background

Globally, the number of HIV-infected people receiving antiretroviral therapy (ART) was about 14.9 million by end of 2014, most of them living in low- and middle-income countries [1–3]. To ensure the sustainability of ART programs in resource-limited settings, it is essential to find effective ways to maintain patients on first-line regimens as long as possible as adherence to ART has a major influence on treatment failure and the development of HIV drug resistance [1, 4–6]. However, the provision of ART in low-resource settings remains challenging due to the shortage of human resources [3]. The World Health Organization (WHO) and the United States President’s Emergency Plan for AIDS Relief (PEPFAR) advocated a strategy to mobilize the involvement of people living with HIV (PLHIV) through task shifting among health workforce team [7]. WHO/UNAIDS proposed the “Treatment 2.0” initiative that aimed to reach and sustain universal access to treatment and capitalize on the preventive benefit of ART through five high-priority goals: 1) optimize drug regimens; 2) provide point of care diagnosis; 3) reduce costs; 4) adapt delivery systems and 5) mobilize communities [3, 8].

In sub-Saharan Africa, peer support and home-based care have become part of the HIV comprehensive care and treatment package [9–11], in which the role of peer support is acknowledged as an essential activity for treatment success [12]. A recent randomized controlled trial (RCT) in Uganda showed that community-based peer health worker intervention had an effect on reducing virologic failure risk after 96 weeks of treatment [13]. A meta-analysis review indicates that peer education programs in developing countries are moderately effective at improving behavioral outcomes but show no significant impact on biological outcomes [14]. On the other hand, in most Asian countries, where the HIV epidemic is in a concentrated stage targeting the high risk population such as injecting drug users (IDUs) and sex workers, the adherence support may pose different challenges [15]. Hence the impact of peer support in Asian countries on virologic failure has not been proven.

In Vietnam, the ART programs have been rapidly scaling up with the support from PEPFAR and Global Fund to fight AIDS, Tuberculosis and Malaria (GFATM). By the end of 2010, more than 82,700 people had access to free ART (70% coverage) [16]. According to the revised National Guidelines [17], VL testing is indicated only for assessing patients suspected of failing the first-line treatment [18, 19]. A qualitative study conducted among 48 PLHIV about adherence obstacles encountered during ART, methods that patients used to enhance adherence, treatment support structures, and attitudes toward home delivery of ART showed that stigma was identified as a strong barrier to ART adherence and that patients seeked more community-based support, preferably from PLHIV who had received sufficient training. Home delivery of ART medications was seen as undesirable by PLHIV, who feared that it might increase stigma and discrimination [20]. We have shown that the community-based peer support had an impact on reducing stigma and discrimination, increasing the access to counseling and testing, improving the quality of life, and enhancing adherence to ART among ART patients presenting at clinical stages 3 and 4 at baseline. However, communitybased peer support had no impact on quality of life among ART patients enrolled at clinical stages 1 and 2 [21]. However, the impact of peer support on treatment outcomes, especially on virologic failure, has not yet been assessed.

We conducted this study with the aim of testing the hypothesis that peer support intervention has an impact on virologic and immunologic responses in HIV-infected patients initiated on first-line ART regimens in a rural resource-limited setting in Quang Ninh, Vietnam. This approach will result in evidence-based ART strategies for large populations in low income and low prevalence settings, which will have an impact on HIV treatment guidelines globally.

## Methods

### Study setting

Quang Ninh province is located along northeastern Vietnam with a population of 1.1 million and an area of 6100 km^2^. Quang Ninh has 14 cities/districts, in which Ha Long City is the biggest (20 communes, 221,000 habitants). It is also home to the famous World Heritage Site, Ha Long Bay. Coal mining, fisheries, and tourism are the main industries.

HIV prevalence in Quang Ninh was about 1%, of which 55% of PLHIV were IDUs (reported by Provincial AIDS Committee-2006). Patients were recruited from four outpatient clinics (OPC): Ha Long CDC-LifeGap clinic, located in the provincial hospital in Ha Long City and supported by PEPFAR which has more resources, and three Global Fund supported clinics (Uong Bi, Ha Long Health Center and Yen Hung). These were the only clinics in the districts and hence there was a low risk for selection bias from both patients and the project.

The cluster randomized controlled trial “Directly Observed Therapy for Antiretrovirals” (DOTARV), registration number NCT01433601 was carried out in 4 districts/cities (Ha Long, Uong Bi, Dong Trieu, Yen Hung). The reasons for choosing these 4 districts/cities were: (i) community based care was not available, (ii) these were adjacent districts, and (iii) each had high HIV prevalence. To minimize contamination (that the effect of peer support to patients in the intervention group would also influence the control groups due to personal relationships among patients in intervention and control group), the unit of randomization and analysis was the cluster (commune).

### Participant recruitment and study procedures

This trial was conducted between July 2007 through November 2011, with 2 years of patient recruitment and 2 years of follow-up.

According to the National Guidelines (2005) [22], HIV-infected individuals were eligible to be registered for free at an ART clinic if they: i) were confirmed HIV positive, ii) possessed valid civil registration information (home address and telephone), iii) had a family member who could act as a supporter, and iv) agreed to enroll and to be followed up in the ART program. Each clinic was staffed with two medical doctors, one adherence counselor, one reception nurse, one phlebotomy nurse, one pharmacist, and one volunteer who was a person living with HIV (PLHIV). The staff were trained on basic and advanced HIV care and treatment and certified by Ministry of Health.

All registered patients received a set standard of care, co-trimoxazole prophylaxis, and were assessed at baseline for socio-economic situations. They were also evaluated clinically, undergoing WHO HIV clinical staging, as well as screening for tuberculosis (TB), viral hepatitis B and C, and opportunistic infections (OIs). Their CD4 counts were taken, and those who were eligible for ART (clinical stage 4 with CD4 count <200 cells/μl or clinical stage 3 with CD4 count <350 cells/μl) were put on the waiting list for ART and selected on a “first come, first served” basis. Patients diagnosed with OIs were treated by OI medications. If TB was diagnosed, patients were referred to the provincial TB hospital in Ha Long City. They were initiated on ART after receiving two months of intensive TB treatment. Every month, a range of 15 to 20 patients per clinic, from both groups, were selected, based on the “first come, first served” recruitment list, to attend a pre-ART readiness training on both an individual and group basis. Each clinic received a quota of patients that were planned to initiate ART stipulated by the local health authorities. The training included HIV basics, stigma and discrimination, positive living, transmission prevention, ART regimens and treatment adherence. A family member also attended the training and became an adherence supporter for the patient. ARV drugs were provided in pre-packed dosage form for convenience and to promote adherence. The first-line ARV regimens included two nucleoside reverse transcriptase inhibitors (NRTIs): stavudine (d4T) or zidovudine (AZT) plus lamivudine (3TC) combined with one non-nucleoside reverse transcriptase inhibitor NNRTI: nevirapine (NVP) or efavirenz (EFV). All care and medications were provided free of charge.

Patients who participated in the study were selected from the pre-ART waiting list. All patients fulfilling the criteria and planned for ART were asked for their consent to take part in the trial. The patients were informed about the trial by study doctors. The patients, if they agreed to participate, signed the informed consent form. Study doctors would assign patients to either the intervention group or the control group based on the commune where they were living from one of a total of 71 communes in 4 districts. The ratio for intervention: control communes was 36:35 (1:1). The clusters in all four districts were randomized by a statistician who was not involved in the study and were matched according to the following criteria (i) urban vs. rural (official registration, based on population density), (ii) vicinity to the clinic (more or less than 5 km), and (iii) population (more or less than 25,000). The randomization of matched clusters was through a computer software by a statistician not directly involved in the project with no local acquaintance. This study followed an open label cluster randomized controlled trial design.

***Inclusion criteria were*** (i) confirmed HIV-infected, (ii) reported as ART-naïve, (iii) resident in any of the four study districts, (iv) age 18 years or older, (v) eligible for ART according to the National Guidelines (2005), CD4 count <200 cells/μl or clinical stage 4, or clinical stage 3 with CD4 count <350 cells/μl, (vi) willing to submit to follow ups and to receive adherence support by a peer-supporter, and (vii) signed a written informed consent.

***Exclusion criteria were*** (i) pregnancy or (ii) mental illness.

### Intervention strategy: peer-support

The peer-support intervention strategy was home-based adherence counseling conducted by peer supporters who were HIV-infected individuals on ART and nominated by fellow patients and health care staff at each clinic site. The qualifications needed for peer-supporters were (i) social ability, (ii) good ART adherence for at least 6 months, (iii) high school graduation, (iv) willingness to participate in the study and (v) passed the qualifying test after the training. The OPC’s and local health authorities proposed candidates that fulfilled the selection criteria’s. The proportion of the peer-supporters to the number of recruited patients living in each district was about 1:20, meaning that one supporter would support a maximum of 20 patients. The peer-supporters received 1-week’s training conducted by project researchers on basic HIV care and support, communication and counseling skills, and the process for completing the adherence checklist form. Two 1-week refresher trainings were provided yearly throughout the study.

The standard support performed by peer-supporters included home-based visits and completion of the adherence checklist form, in which patients were asked about their wellbeing, OI and adverse drug reaction (ADR) signs and symptoms, the times at which they took the pills, any doses missed for last 4 days, barriers to adherence, and pill count. If an incomplete adherence was reported, the peer supporter would counsel and discuss with the patients and family supporters how to improve the adherence.

The initial schedule of support was twice a week in the first 2 months, then once a week when patients’ adherence passed assessments. Additional visits were provided if patients were sick or had a serious ADR or a history of poor adherence. A telephone call was used to arrange a 15- to 30-min appointment place in advance between peer supporters and patients to minimize wasting time or to ensure confidentiality for patients who feared disclosure of their HIV status to others in their surroundings. Due to the associated stigma, the supporters did not wear a work outfit for home visits to minimize the patient’s fear of stigma developing from others in their surroundings. Before being recruited for the study, patients were made aware that being a part of the study would require them to disclose their HIV status. They were warned about the possibility of stigmatization. If patients were not comfortable with that possibility or if they experienced any harm during the study, patients would choose not to be a part of the study. This was monitored during the OPC visits. To ensure satisfactory work by the peer supporters, bi-weekly supervision of peer-support activities was conducted by a peer-support group leader in each district. Monthly supervision meetings of peer-support activities were performed by project researchers.

Patients in both intervention and control groups received a set standard of care and treatment according to the National Guidelines [22] including pre-ART initiation, which consisted of three ART adherence training sessions in both individual and group settings. Health checks, blood sampling and drug dispensations were carried out on a monthly basis at the OPC. Selfreported adherence for the last-4-day period was assessed quarterly by an adherence counseling staff member. CD4 counts were run at baseline and every 6 months by using the Partec CyFlow® system in Uong Bi hospital and the Becton Dickinson® system in Quang Ninh provincial hospital.

### Viral load and CD4 count screening

The Exavir™ Load, an enzyme-linked immune-sorbent assay (ELISA)-based VL quantification test (Cavidi AB, Uppsala, Sweden [23, 24]), was used in Vietnam for the first time to monitor the virologic outcomes for all participants every 6 months for 24 months of the study. The detection limits range from 200 to 410,000 copies/ml [25]. Details on the laboratory procedures of ExaVir Load assay were reported in an earlier publication [26].

CD4 counts were run at baseline and every 6 months by the Partec CyFlow® in Uong Bi hospital and the Becton Dickinson® in Quang Ninh provincial hospital.

### Adherence assessment

In both the intervention and control groups, patients were assessed by health care staff at the clinic for adherence every 3 months using an adherence checklist modified from the contextualized Adult AIDS Clinical Trials Group (AACTG) adherence instrument [27]. In this checklist, patients self-reported any OI and ADR symptoms, whether they had missed any doses during the last 4 days, or if they had incorrectly measured their pill-count.

*Incomplete adherence* was defined as the patient stating in the 3-month adherence checklist form that he or she missed more than one dose (2 or more; either in morning or evening) of ARV during the 24 months of their follow-up time.

*Complete adherence* was defined as the patient self-reporting in the 3-month adherence checklist form that he or she did not miss any dose or just one dose (either in the morning or evening) of ARV drugs during the 24 months of their follow-up time.

### Definitions

*Virologic failure* was defined as either (i) primary virologic failure if VL >1000 copies/ml after 6 months of ART initiation or (ii) secondary virologic failure if VL was undetectable (<200 copies/ml) after 6 months of ART initiation and then became >1000 copies/ml at any time point during the follow-up. Virologic failure patients were reported to the attending medical doctors and adherence counselors in their respective clinics. Then a follow-up VL test was repeated after at least one month but within 3 months of the initial virologic failure. If VL remained >1000 copies/ml, the patient was then reported to an OPC doctor and flagged for a confirmatory PCR VL. According to VGHADT (2009) [17], patients were eligible for switching to second-line therapy if they meet the criteria of clinical or immunologic failure and, if available, they have been confirmed to have a PCR VL >5000 copies/ml. Additionally, patients with detectable viral load in the intervention group received an intensive adherence counseling support by the peer supporters through the provision of two to three home visits per week.

*Death* events were ascertained by a medical doctor confirmation or, in the intervention group, by a peer-supporter through telephone calls or home visits. In cases of a missing follow-up, event of death was confirmed through telephone calls to family members and home visits by peer supporters.

*Lost-to-follow-up* was defined as: when the patient was either arrested or placed in a compulsory drug rehabilitation center for 24 months due to active heroin use, thus disabling follow-up during the study period; or when the patient did not show up at the OPC for 3 consecutive visits; or if the patient voluntarily withdrew from the study. Patients in both groups were called to determine the reason of their lost-to-follow-up.

*Transferred* patients were defined as those who were confirmed as being registered with another OPC which was outside of our four study sites.

*Changed-regimen* was defined as a patient who had to change one of the three ARV drugs in the regimen for any clinical reason (ADRs or TB co-infection treatment) during ART.

### Study endpoints

The primary endpoint was the risk of virologic failure during 24 months of follow-up.

The secondary endpoint was the time course of CD4 cell counts over the period of 24 months of follow up.

### Data collection

Data were collected as follows: i) socio-economic situation at baseline, ii) clinical and laboratory data at baseline and follow-up visits every 6 months, (iii) self-reported adherence form was administered by health staff every 3 months, and (iv) ART adherence forms completed by peer supporters.

### Sample size

Patients were allocated to the intervention group according to a randomization of clusters (communes) where patients lived. Sample size for cluster randomized trial was calculated by using formulas in [28]. Assuming the baseline figure for virologic failure risk in the intervention group equal to 5%, and equal to 15% in the control group, a power of 80%, the significance level of the two-sided alpha 0.05, a randomization ratio of 1 (intervention):1 (control), an estimation of intraclass coefficient equal to 0.1, a number of clusters in each group equal to 35 and adding 30% for lost-to-follow-up, the minimum needed sample size was 638 patients (about 319 patients per study arm).

### Statistical analysis

Intention-to-treat analysis was used to calculate virologic failure risk.

Mean, median and standard deviation were used for summarizing numerical variables, frequencies, and percentages for the categorical variables.

Chi-square tests were performed to compare intervention and control groups for demographic and clinical characteristics at baseline.

Crude relative risks (RR) at 6, 12, 18, and 24 months were calculated to assess the relationship between virologic failure and intervention/control groups at different time points.

Kaplan-Meier curves were produced, together with a log-rank test, to compare time to virologic failure between control and intervention group. Cox proportional hazards frailty model, adjusting for potential confounders, was used to analyze the hazard rate among the intervention and control groups, taking into account the clustered nature of the data. Schoenfeld residuals were used for checking the proportional hazard assumptions; no timedependent variables were considered.

The final model was selected using a forward- stepwise selection with a *p*-value cut-off for entering the model equal to 0.1. A likelihood-ratio test was used for testing the null hypothesis of no variance of the frailty effects.

CD4 count trends over time were analyzed using a mixed-effects model with a polynomial function of time in the fixed component. Due to the hierarchical structure of the data, random effects of clusters (communes, individual and measurement) were incorporated into the model. Square-root transformation was used for the CD4 count approximating a normal distribution [29].

Once the data was collected, the models were adjusted for the following variables: randomized groups (control vs intervention); age (≥35 vs <35 years); gender (male vs female); WHO clinical stage (stage 1 and 2 vs stage 3 and 4); baseline VL (≥100,000 copies/ml vs <100,000 copies/ml) and baseline CD4 counts (≥100 cells/μl vs <100 cells/μl); ART-naïve status (yes vs no); history of IDU (yes vs no); TB history (yes vs no); history of OIs (yes vs no); having an HIV-infected family member (yes vs no); receiving ART in Halong CDC clinic (yes vs no), changed ART regimen (yes vs no); and incomplete adherence (yes vs no). Since there was a significant difference between the groups receiving ART in Halong CDC clinic, it was especially important to adjust for this difference.

The above mentioned demographic and clinical characteristics at baseline were tested as independent variables both in the survival and mixed effects models. *P*-values less than 0.05 were considered significant in the final model. The statistical analyses were performed using STATA version 12.0 (College Station, StataCorp LP, TX, USA).

## Results recruitment of the cohort

During the period of July 2007 through November 2009, a total of 640 HIV-infected participants (332 intervention patients and 308 control patients) were enrolled from 59 communes (30 intervention communes and 29 control communes). As 12 of the communes had no participants, these were removed from the analysis. Ha Long City had the majority of patients (418; 65%), then Uong Bi (87; 14%), Dong Trieu (71; 11%) and Yen Hung (64; 10%). The distribution of patients in the four study clinics were: Ha Long CDC Life-Gap clinic (307; 48%); The Uong Bi Hospital clinic included patients from Dong Trieu district (181; 28%); Ha Long Health Center clinic (106; 17%) and Yen Hung clinic (46; 7%). On average, each commune had 11 patients. However, the number of patients was not distributed equally among communes: the Cao Xanh commune in Ha Long City has the highest number of patients (46) while 12 communes had only 1 patient each and another 12 had no patients enrolled in the study (Table 1). A sensitivity analysis was done and found that the inclusion of the 12 communes with 1 patient each did not invalidate the findings of the study (data not shown).

**Table 1:** Number of patients in each cluster enrolled in either the intervention or control groups

Intervention communes	Nr patients	Control communes	Nr patients
Yet Kieu	16	Tran Hung Dao	30
Bach Dang	25	Hon Gai	26
Cao Xanh	46	Hung Thang	6
Hong Hai	26	Cao Thang	32
Bai Chay	23	Ha Lam	42
Dai Yen	7	Ha Tu	23
Ha Khanh	13	Ha Khau	20
Ha Phong	13	Hong Ha	21
Tuan Chau	7	Viet Hung	9
Gieng Day	15	Ha Trung	18
Thanh Son	16	Bac Son	4
Quang Trung	21	Phuong Dong	8
Phuong Nam	3	Dien Cong	1
Vang Danh	10	Trung Vuong	13
Thuong Yen Cong	1	Nam Khe	4
Mao Khe	44	Yen Thanh	6
Yen Duc	1	Dong Trieu	3
Kim Son	4	Tan Viet	1
Hung Dao	1	Thuy An	1
Xuan Son	1	Yen Tho	4
Binh Khe	1	Hoang Que	6
Viet Dan	1	Hiep Hoa	2
Binh Duong	3	Lien Vi	4
Quang Yen	8	Cong Hoa	8
Yen Giang	2	Ha An	5
Hoang Tan	1	Cam La	1
Yen Hai	4	Dong Mai	1
Minh Thanh	6	Phong Hai	6
Nam Hoa	4	Phong Coc	3
Lien Hoa	9		0
Total (I & C)	332		308
Total nr patients		640	

After conducting a one-week pilot training session, we selected 14 qualified peer supporters (8 females, 6 males), aged between 25 and 44 years. The number of supporters was proportional to the number of intervention patients in each district/city. Seven peer supporters were based in Ha Long (191 patients), 3 in Uong Bi (51 patients), 2 in Dong Trieu (56 patients) and 2 in Yen Hung (34 patients). Each peer supporter was responsible for visiting between 10 and 20 patients.

**Fig. 1:**
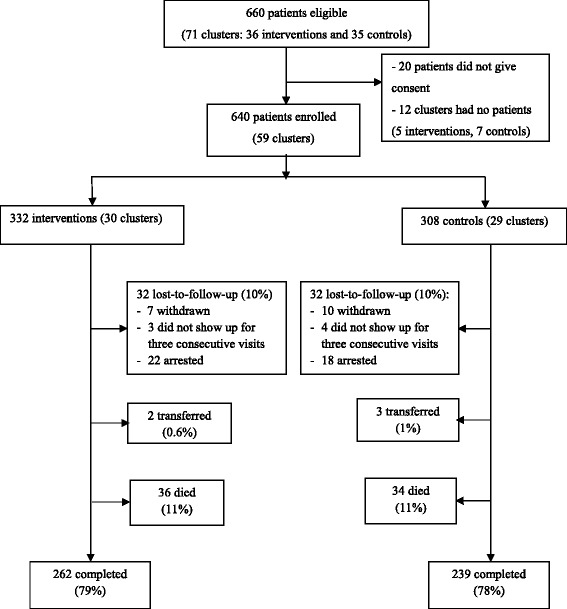
Patient recruitment and outcome status after 24 months of follow-up

By the end of the study, mean follow-up time was 20.4 ± 7.2 months (20.5 ± 7.2 and 20.4 ± 7.3 months for intervention and control groups, respectively), 78% (501/640) of patients remained on ART, 11% (70/640) were dead, 10% (64/640) were lost-to-follow-up and 1% (5/640) had transferred to other clinics. Among the lost-to-follow-up patients, 17 (27%) had voluntarily withdrawn, 7 (11%) did not show up for three consecutive visits and 40 (62%) were arrested and put in rehabilitation centers due to injecting heroin. Eleven patients were arrested during the ART treatment, however, they had continuous access to ART and then resumed their ART at the clinics after being released from the rehabilitation center, therefore they were not considered lost-to-follow-up. The distribution of retention in care, dead, lostto-follow-up, and transferred patients were equally distributed in both intervention and control groups (Fig. 1).

Table 2 described the characteristics of the cohort divided by control and intervention groups at baseline with no significant differences observed between the two groups, apart from in those patients from the Ha Long CDC clinic.

**Table 2:** Demographic and clinical characteristics of 640 patients enrolled in both the intervention and control groups

Variables		Total (*n* = 640)	Control (*n* = 308)	Intervention (*n* = 332)	*P*-value**
		*n* (%)	*n* (%)	*n* (%)	
Age	Median (IQR)	31.9 (28.2–35.1)	31.7 (28.2–34.5)	32.1 (28.4–35.6)	
	<35 years old	474 (74.1)	235 (76.3)	239 (72.0)	0.21
	≥35 years old	166 (25.9)	73 (23.7)	93 (28.0)	
Sex	Male	452 (70.6)	216 (70.1)	236 (71.1)	0.79
	Female	188 (29.4)	92 (29.9)	96 (28.9)	
Marital status	Single	195 (30.5)	89 (28.9)	106 (32.0)	0.44
	Married/divorced	445 (69.5)	219 (71.7)	226 (68.1)	
ART-naïve status	Naïve	606 (94.7)	289 (45.2)	317 (49.5)	0.35
	Non-naïve	35 (6.0)	19 (6.2)	16 (4.8)	
History of OIs*	Yes	182 (28.4)	91 (29.5)	91 (27.4)	0.55
	No	458 (71.6)	217 (70.5)	241 (72.6)	
Occupation	Employed	493 (77.0)	243 (78.9)	250 (75.3)	0.31
	Unemployed	147 (23.0)	65 (21.1)	82 (24.7)	
Time to be known infected ≥6 months	<6 months HIV-	156 (24.4)	69 (22.4)	87 (26.2)	0.31
		449 (70.0)	220 (71.0)	229 (69.0)	
HIV transmission route (self-reported)	IV Drug use	297 (46.4)	136 (44.2)	161 (48.5)	0.27
	Sexual and others	343 (53.6)	172 (55.8)	171 (51.5)	
History of IDU	Yes	337 (52.7)	151 (49.0)	186 (56.0)	0.08
	No	303 (47.3)	157 (51.0)	146 (44.0)	
Viral hepatitis	Yes	207 (33.7)	92 (31.1)	115 (36.2)	0.18
	No	407 (36.3)	204 (68.9)	203 (63.8)	
History of TB	Yes treatment	99 (15.5)	53 (17.2)	46 (13.9)	0.24
	No	541 (84.5)	255 (82.8)	286 (86.1)	
Having an HIV infected family member	Yes	256 (40.0)	132 (42.9)	124 (37.3)	0.09
	No	384 (60)	176 (57.1)	208 (62.7)	
WHO clinical stage	Clinical stage 1 or 2	298 (46.6)	142 (46.1)	156 (47.0)	0.82
	Clinical stage 3 or 4	342 (53.4)	166 (53.9)	176 (53.0)	
BMI”	18+ kg/m2	409 (64.0)	188 (61.0)	221 (66.7)	0.15
	<18 kg/m2	231 (36.0)	120 (39.0)	111 (33.3)	
Hemoglobin level	<100 g/L	73 (11.4)	33 (10.7)	40 (12.0)	0.61
	≥100 g/L	520 (81.2)	253 (82.1)	267 (80.4)	
CD4 counts	Median (IQR)	83 (29–176)	82 (27–183)	84 (30–168)	0.90
	<100 cells/μl	359 (56.1)	172 (55.8)	187 (56.3)	
	>100 cells/μl	281 (43.9)	136 (44.2)	145 (43.7)	
VL at baseline (copies/ml)	<100.000	426 (66.7)	209 (67.9)	217 (65.6)	0.54
	≥100,000	213 (33.3)	99 (32.1)	114 (34.4)	
regimen	D4T/3TC/NVP ART^	533 (83.3)	258 (83.8)	275 (82.8)	0.75
	Other regimens	107 (16.7)	50 (16.2)	57 (17.2)	
Clinics	Halong CDC	307 (48.0)	168 (54.5)	139 (41.9)	**0.001**
	Other clinics	333 (52.0)	140 (45.5)	193 (58.1)	

*OIs opportunistic infections, *IDU* Injecting drug use, *TB* tuberculosis, *BMI* Body Mass Index, *ART* Antiretroviral therapy, *VL* viral load

**Chi-square test

### Clinical outcome

At month 24, clinical outcomes improved, as mean body weight increased from 50.2 ± 7.3 kg at baseline to 53.7 ± 7.9 kg at month 24 (*p* < 0.001). However, there was no significant difference in gaining weights between the intervention and control groups (50.2 ± 6.8 vs 50.3 ± 7.7 kg at baseline, respectively; *p* = 0.86 and 53.5 ± 7.0 vs 53.9 ± 8.8 kg at month 24, respectively; *p* =0.66). There were 163 (25%) patients who had to change their treatment regimens due to ADRs, of which the most common reasons for changing the regimens were peripheral neuropathy (104; 64%), rash (25; 15%), hepatitis (15; 9%) or TB treatment (9; 6%). However, there was no significant difference between the intervention and control groups (89; 27% vs 74; 24%, *p* = 0.47). Fifty-four patients (8.5%) developed at least one OI or TB after 6 months of ART (8% in the intervention group and 9% in the control). Six (1%) patients switched to second-line regimens (3 in the intervention group and 3 in the control); all of these switched after treatment and at least 18 months after having confirmatory PCR VL >5000 copies/ml. The overall mortality risk was 11% with no significant difference between the intervention and control group (results not shown). Based on the monitoring during OPC visits no case of peer-support induced stigmatization was detected during the study.

### Virologic failure

**Fig. 2:**
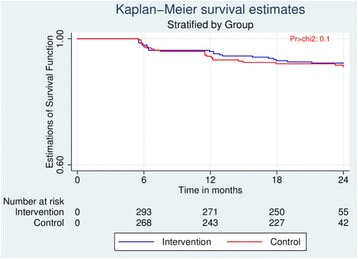
Log-rank test for equality of survival curves between intervention and control group (Log-rank *p* = 0.77)

After 24 months of ART initiation, a total of 46 patients (7.2%) experienced virologic failure. There was no significant difference regarding virologic failure risks between the intervention (cumulative virologic failure at 24 months equal to 23/332; 6.9%) and control groups (23/308, 7.5%) at any time point (6, 12, 18, or 24 months) (Table 3). Of the 46 virologic failure cases, 22 (48%) were primary virologic failure. The Kaplan-Meier curves showed no significant difference in time to virologic failure between the intervention and control group (Log-rank *p*-value = 0.77) (Fig. 2).

**Table 3:** Impact of peer support on virologic failure at months 6, 12, 18 and 24 (*n* = 640)

Months	Intervention group (*n* = 332)		Control group (*n* = 308)		RR	95% CI	*P*-Value**
	Virologic failure risk (95% CI)	Virologic failure risk (95% CI)	Virologic failure risk (95% CI)	Cumulative virologic failure risk (95% CI)			
6 m	11 (3.3%, 1.4–5.2%)	11 (3.3%, 1.4–5.2%)	11 (3.5%, 1.5–5.6%)	11 (3.5%, 1.5–5.6%)	0.9	0.4 < RR < 2.1	0.86
12 m	6 (1.8%, 0.3–3.2%)	17 (5.1%, 2.7–7.5%)	9 (2.9%, 1–4.8%)	20 (6.5%, 3.7–9.3)	0.6	0.2 < RR < 1.7	0.35
18 m	4 (1.2%, 0.1–2.3%)	21 (6.3%, 3.6–9%)	1 (0.3%, 0–1%)	21 (6.8%, 3.9–9.7%)	3.7	0.4 < RR < 33	0.21
24 m	2 (0.6%, 0–1.4%)	23 (6.9%, 4.1–9.7%)	2 (0.6%, 0–1.5%)	23 (7.5%, 4.4–10.5%)	0.9	0.1 < RR < 6.5	0.94

*CI* Confidence Interval, *RR* Relative Risk

**Chi-square test

The adjusted results from the Cox proportional hazards frailty model showed that the significant risk factors for developing virologic failure were ART-non-naïve status [aHR 7.0;(95% CI 3.3–14.7); *p* < 0.01]; baseline VL ≥100,000 copies/ml [aHR 2.3;(95% CI: 1.2–4.3); *p* < 0.05], and incomplete adherence [aHR 3.1;(95% CI: 1.1–8.9); *p* < 0.05] (Table 4).

**Table 4:** Virologic failure risk analyzed with bivariate and adjusted HRs of Cox proportional hazards frailty model

	Bivariate		Adjusted	
Characteristics	HR (95% CI)	*p*-value	aHR (95% CI)	*p*-value
Intervention group	1.0 (0.5;1.7)	0.94		
Male gender	2.0 (0.1;4.0)	0.05		
Age <35 years	0.7 (0.4;1.4)	0.37		
Severe HIV (clinical stage 3 or 4)	1.2 (0.6;2.1)	0.59		
**History of IDU**	**1.9 (1.0;3.3)**	**0.04**		
**ART-non-naïve status**	**4.8 (2.4;9.4)**	**<0.01**	**7.0 (3.3–14.7)**	**<0.01**
TB history	1.6 (0.8;3.2)	0.15		
**VL at baseline ≥100,000 copies/ml**	**1.6 (0.9;2.8)**	**0.13**	**2.3 (1.2–4.3)**	**<0.05**
CD4 at baseline < 100 cells/μl	1.4 (0.8;2.5)	0.28		
Having an HIV-infected family member	0.5 (0.3;0.9)	0.03		
From Ha long OPC	1.0 (0.6;1.8)	0.91		
History of OIs	0.5 (0.2;1.0)	0.06		
Changed ART regimen	1.1 (0.5;−2.0)	0.85		
**Incomplete adherence**	**2.4 (0.9;6.7)**	**0.09**	**3.1 (1.1–8.9)**	**<0.05**

*OIs* opportunistic infections, *VL* viral load, *IDU* Injecting drug use, *TB* tuberculosis, *ART* Antiretroviral therapy, *OPC* outpatient clinic, *HR* Hazard Ratio, *aHR* adjusted Hazard Ratio

The boldface indicates significant (p<0,05) result

There was no significant effect of intervention on time to virologic failure.

After excluding the 35 ART-non-naïve patients, the statistical analysis among only 605 naïve patients (316 in the intervention group and 289 in the control group) also showed no significant difference in virologic failure risks between intervention and control group [6.3% vs 5.2%; respectively, RR = 1.22; (95% CI: 0.63–2.37); *p* = 0.56] (data not shown).

### Immunologic outcome

**Fig. 3:**
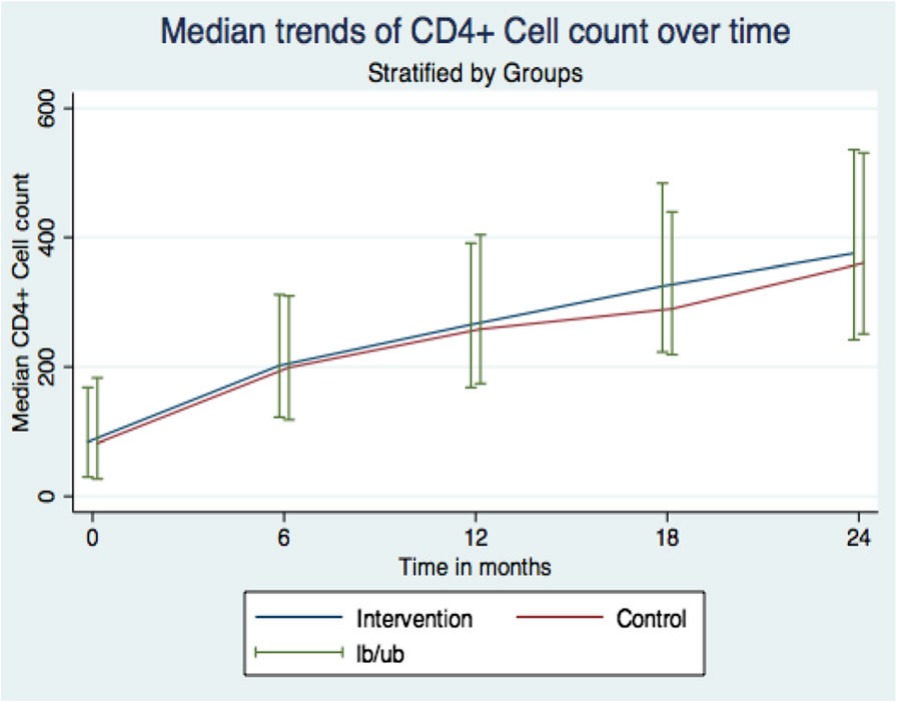
Median trends of CD4 counts over time between intervention and control groups

The median CD4 counts increased rapidly from 83 cells/μl (IQR 29–176) at baseline to 202 cells/μl (IQR 121–311) at month 6, to 260 cells/μl (IQR 168–400) at month 12, to 305 cells/μl (IQR 220–463) at month 18 and to 371 cells/μl (IQR 249–534) at month 24 (Fig. 3). The increase of median CD4 count from baseline to month 24 was 286 cells/μl (292 cells/μl in the intervention group and 279 cells/μl in the control group).

Patients with baseline VL ≥100,000 copies/ml [adj.sq.coeff. (95% CI): −0.9 (−1.5;−0.3); *p* < 0.01] and baseline CD4 count <100 cells/μl [adj.sq.coeff. (95% CI): −5.7 (−6.3;−5.1); *p* < 0.01] had a significantly lower increase of CD4 count compared to the other patients. On the other hand, patients with an HIV-infected family member had a significantly higher increase in CD4 count compared to those who did not have an HIV-infected family member [adj.sq.coeff. (95% CI): 1.3 (0.8;1.9); *p* < 0.01] (Tables 4 and 5).

**Table 5:** Effect of predictors and evolution of CD4 cell count on HIV infected patients on ART

	Bivariate		Adjusted	
Characteristics	Coeff (95% CI)	*p*-value	Adj.sq.Coeff (95% CI)	*p*-value
Intervention group	0.2 (−0.6;−0.9)	0.69		
Female gender	−3.0 (−3.8;−2.3)	<0.001		
Age <35 years	−0.3 (−1.1;−0.6)	0.54		
Severe HIV (clinical stage 3 or 4)	−2.1 (−2.8;−1.4)	<0.001		
History of IDU	−2.1 (−2.8;−1.4)	<0.001		
ART-non-naïve status	0.8 (−0.8;2.5)	0.32		
TB history	−0.2 (−1.2;0.8)	0.68		
**VL at baseline ≥100,000 copies/ml**	**−2.8 (−3.6;−2.0)**	**<0.001**	**−0.9 (−1.5;−0.3)**	**<0.01**
**CD4 at baseline <100 cells/μl**	**−6.8 (−7.4;−6.3)**	**<0.001**	**−5.7 (−6.3;−5.1)**	**<0.001**
**Having an HIV-infected family member**	**2.5 (1.8;3.3)**	**<0.001**	**1.3 (0.8;1.9)**	**<0.001**
From Ha long CDC OPC	−0.5 (−1.2;0.3)	0.22		
History of OIs	1.1 (0.3;1.9)	<0.01		
Changed ART regimen	1.0 (0.1;1.8)	<0.05		
Incomplete adherence (missing more than one doses in 24 months)	−1.3 (−3.1;0.5)	0.15		
Month	0.4 (0.4;0.4)	<0.001	0.39 (0.38;0.41)	<0.001

*OIs* opportunistic infections, *VL* viral load, *IDU* Injecting drug use, *TB* tuberculosis, *ART* Antiretroviral therapy, *OPC* outpatient clinic, *Coeff* Coefficient, *adj.sq.Coeff* adjusted square Coefficient

The results showed no significant effect of the intervention on the evolution of CD4 count trend between the intervention and control groups.

## Discussion virologic failure, mortality and retention rates

To our knowledge, this is the first randomized controlled trial study in Vietnam to assess the impact of peer support on virologic and CD4 outcomes among HIV-infected patients starting ART. Our study showed a comparably low virologic failure risk, low mortality and high retention rate after 24 months of follow-up. This indicates that a well-funded and organized ART program implemented through PEPFAR and Global Fund in Vietnam can be rolled out successfully in remote and resource-limited settings.

After 24 months follow-up, the virologic failure risk in our study (7.2%) was lower than anticipated when the study was planned and compared to other countries (15 to 20%) [30–33]. This, in retrospect, may have made the study underpowered. Among the 46 virologic failure cases in our study, 22 (48%) were secondary virologic failure (10 in the intervention group and 12 in the control group). In Sub-Saharan African countries, findings from systematic reviews showed an overall virologic failure risk (VL >1000 copies/ml) was 24% within 12 months of ART [34] and 15% after 24 months of ART [34], the highest risk (43%) was seen in a Rwanda [35].

In our study, the proportion of virologically suppressed patients on ART at 24 months was 94% which is higher than those reported from other resource-constrained settings including Uganda (86%) [36], Malawi (84%) [37] and Cameroon (52%) [38]. Studies in Vietnam reported that the proportion of patients with viral suppression among IDU populations were 73% [39] and 70% in Ho Chi Minh City [18]. In neighboring Cambodia, 88% (306/346) of patients had achieved VL <400 copies/ml [40] and in Thailand, 15% (55/345) had virologic failure (>400 copies/ml) at 24 months [41]. However, most of these studies reported ontreatment results that were analyzed in a cross-sectional fashion and included both ARTnaïve and non-naïve patients. The good treatment outcome in our study may be explained by several factors including that the vast majority of patients were ART-naïve and the VL results were reported to the treating doctors since knowledge about viremia may result in intensification of the adherence support, both for intervention and control group patients.

Despite the fact that the majority of the patients were severely immune-suppressed with low CD4 counts at baseline and half were reported to be at clinical stage 3 or 4, our study showed a lower mortality risk (11% or 6.4/100 person-years) compared to other studies in resourcelimited countries [36–38, 42–44]. However, the mortality risk of this study was higher than that of studies in middle- and high-income countries [45–47]. The survival and causes of death among patients in our cohort was previously reported [48].

The retention rate of patients on ART was 78% after 24 months with no significant difference between the intervention and control groups (*p* = 0.7). High retention rates in care indicate not only an improved health care system and ART programs in Vietnam, but also effective care and support activities in the community to motivate and engage patients in care. A recent study in Vietnam conducted among 4531 adults and 313 children showed that 81.2 and 84.4%, respectively, were still on ART after 12 months [49]. This result was similar with the retention rate in Thailand (80.8% after 5 years) [45], Cambodia (80% after 4 years) [43] and higher compared to studies in sub-Saharan Africa with 24 months of follow-up: Uganda (72%) [36] and Malawi (66%) [37]. Other studies also showed that engagement of HIV care is associated with improved clinical, virologic and immunologic parameters and survival outcomes [50, 51]. Therefore, retaining HIV-infected patients in care has become a public health issue to ensure the success and sustainability of ART programs [52].

### Immunologic outcomes

As shown in our study, CD4 counts responded well after 24 months of ART (overall increase of 286 cells/μl). This finding is in line with other studies that show how CD4 cell counts increased quickly, in particular after 6 months of ART [38, 53]. However, there was no significant difference between the intervention and control groups in CD4 trends after the adjustment (*p* = 0.69). Factors that predict the increase of CD4 counts were the VL at baseline ≥100,000 copies/ml, a baseline of CD4 counts <100 cells/μl, and having an HIV-infected family member. In a study in Thailand, CD4 count at baseline and changes in CD4 count were important in predicting CD4 counts ≤200 cells/μl [54].

In our study, after 24 months we found 45 patients (7%) had immunologic failure, of whom 23 (6.9%) were in the intervention group and 22 (7.1%) in the control group (no significant difference, *p* >0.05). Also, there was a high discordance (71%) between immunologic and virologic failure. Only 13 patients (29%) had both immunologic failure and virologic failure (*p* <0.001).

### Factors associated with virologic failure

Our study showed that ART-non-naïve status, high baseline VL (≥100,000 copies/ml) and incomplete adherence (missing more than one dose during 24 months) were risk factors for virologic failure. High VL (>100,000 copies/ml) at baseline can be a predictor for the slower increase of CD4 counts and mortality [48]. Therefore, our findings were in line with other studies that high VL at baseline is a predictor for virologic failure and mortality during ART [18, 55, 56]. Contrarily, studies in Thailand shows low baseline CD4 count and race/ethnicity were independent predictors of virologic response, however baseline VL and gender were not [54]. Also, univariate analysis showed having a child was significantly associated with lower virologic failure risk [41].

### Impact of peer support intervention

The study results might answer the research question in that there was no significant difference between the intervention and control groups in both cumulative virologic failure risk and time to virologic failure after 2 years of follow-up. This result could be explained in several ways. While the simplest explanation is that peer support inherently does not affect treatment outcomes, we cannot exclude the possibility that our sample sizes may simply have been insufficiently large. With a larger study, we might have been able to gain enough statistical power to discern a difference between the intervention and control groups. Also, the adherence is self-reported, and may have been affected by observer bias. Also, the ART program in Vietnam has been well funded and implemented through international donors including PEPFAR and Global Fund. Before ART was initiated, each patient had to be assessed for ART readiness, name a supporting family member and attend three adherence counseling sessions according to the National Guidelines [22]. Furthermore, the eventual effect of the intervention might also be masked by a “ceiling effect” in which the control group also received a sufficient baseline adherence support. Free ARV may have also contributed to this “ceiling effect”. The baseline support may have been at such a high level that additional support did not affect treatment outcomes.

In addition, patient support could have originated from a number of different sources that may have contaminated the study including the OPCs and the community-based programs. Quang Ninh province, with a comparably high HIV prevalence, was receiving PEPFAR support in order to set up “comprehensive care, treatment and support” programs with NGOs, which were very active during the time of the project implementation. The availability of community-based activities provided by “the Bright Future” and other community outreach groups might have constituted a “contamination” where patients in the intervention group could meet and share adherence experiences with patients in the control group.

Furthermore, IDU in both the control and intervention groups had incentives to remain on the treatment regimens. Active IDUs may get arrested and placed into a drug rehabilitation centers. The risk of this may be increased if they fail to follow the treatment schedule, and thereby get attention from authorities. IDUs also received further counseling at the OPC and, for those in the intervention group, from the peer supporters.

In our study, 400 (63%) patients lived with their parents and other family members who might also play an important role as “internal supporters” to support patients in taking ARVs. Vietnamese culture strongly values the care of the extended family members, so most patients had good support for adherence from their families.

In our study, we only reported data to the 24-month-follow-up; hence the sustained or longterm effect of the adherence support intervention cannot be excluded. It should be noted that as patients started their ARV and improved their health, their perceived need for peer support may have declined. It was also apparent from discussion with peer supporters and patients that the personal relationship between the patient and supporter was crucial for long term peer support and follow up. Many patients saw the benefit and developed a close bound to their peer supporter, sometimes too close from the peer supporter’s point of view. In other cases, the patients perceived the support as an annoyance. A recent study in Uganda has also shown that peer health care intervention had no impact on cumulative risk of virologic failure and virologic outcomes on short-term ART and suggested that it might be best suited for patients who have taken ART for longer periods, especially as it may mitigate the effects of “treatment fatigue” as patients tire of continually taking ART [13]. Therefore, to assess the sustained effect of the peer support intervention in our cohort, further research to continue following up patients with VL and CD4 count monitoring up to at least 48 months is needed.

Despite the results showing peer support had no significant effect on virologic failure, it is nevertheless clear that good outcomes can be achieved in resource-limited communities. The support the patients received may still be considered a global standard of care.

### Methodological considerations

This is the first cluster randomized controlled trial on HIV conducted in a rural setting in Vietnam. We had some limitations and constraints in logistics, in recruiting patients, collecting data, and analysis due to a lack of precedence to follow. It took 2 years for recruiting enough patients instead of 10 months that we had originally planned. There were 35 (6%) patients who were ART-non-naïve. However, as the study was intention-to-treat and the non-naïve status was revealed after enrollment, these cases were still included in the study and the results might not represent whole treatment-naïve population. Patients in both groups may receive other community-based supports that might be a “contamination”. In addition, patients in the intervention group could meet and share adherence experiences with patients in the control group at the clinic (i.e., there were 2 couples of whom the men in the intervention group married women in control group). Some patients were willing to move or even travel long distances in order to receive treatment from a certain clinic. This devotion to obtaining ARV could have contributed to the high adherence in both groups. The CD4 counts were done in two different hospital laboratories which may bias the estimation of immunologic failure. As the clusters were randomized before the recruitment of patients and the selection process of the patients should have randomized the individual clusters, the difference in the number of patients in each arm is unlikely to lead to bias. A limitation in our study was that the adherence data collected from peer supporters during their patient encounters in the intervention group wasn’t included in the analyses.

## Conclusions

ART programs in resource-limited rural settings like Quang Ninh, Vietnam can provide an effective care and treatment with a low virologic failure risk if the patients are well-prepared for ART and followed up regularly by an out-patient clinic. Peer support to improve adherence did not show any impact on virologic failure and CD4 trends during first 2 years of ART. High VL at baseline is a predictor for virologic failure and CD4 trends.
